# Key Scientific Issues in the Health Risk Assessment of Trichloroethylene

**DOI:** 10.1289/ehp.8690

**Published:** 2006-05-09

**Authors:** Weihsueh A. Chiu, Jane C. Caldwell, Nagalakshmi Keshava, Cheryl Siegel Scott

**Affiliations:** National Center for Environmental Assessment, Office of Research and Development, U.S. Environmental Protection Agency, Washington, DC, USA

**Keywords:** epidemiology, mode of action, peroxisome proliferators, pharmacokinetics, trichloroethylene, risk assessment

## Abstract

Trichloroethylene (TCE) is a common environmental contaminant at hazardous waste sites and in ambient and indoor air. Assessing the human health risks of TCE is challenging because of its inherently complex metabolism and toxicity and the widely varying perspectives on a number of critical scientific issues. Because of this complexity, the U.S. Environmental Protection Agency (EPA) drew upon scientific input and expertise from a wide range of groups and individuals in developing its 2001 draft health risk assessment of TCE. This scientific outreach, which was aimed at engaging a diversity of perspectives rather than developing consensus, culminated in 2000 with 16 state-of-the-science articles published together as an *Environmental Health Perspectives* supplement. Since that time, a substantial amount of new scientific research has been published that is relevant to assessing TCE health risks. Moreover, a number of difficult or controversial scientific issues remain unresolved and are the subject of a scientific consultation with the National Academy of Sciences coordinated by the White House Office of Science and Technology Policy and co-sponsored by a number of federal agencies, including the U.S. EPA. The articles included in this mini-monograph provide a scientific update on the most prominent of these issues: the pharmacokinetics of TCE and its metabolites, mode(s) of action and effects of TCE metabolites, the role of peroxisome proliferator–activated receptor in TCE toxicity, and TCE cancer epidemiology.

Trichloroethylene (TCE; CAS Registry no. 79-01-6) is a chlorinated solvent that has been widely used as a metal degreaser, extractant, and chemical intermediate and is now a common environmental contaminant. TCE has been identified in at least 1,500 hazardous waste sites regulated under the Comprehensive Environmental Response, Compensation, and Liability Act of 1980 (CERCLA 1980) or the Resource Conservation and Recovery Act of 1976 (RCRA 1976). TCE can be released into the atmosphere from vapor degreasing operations, enter surface waters via direct discharges, and enter groundwater through leaching from disposal operations and Superfund sites. In addition, TCE can be released to indoor air from use of consumer products, vapor intrusion from groundwater through underground walls and floors, and volatilization from the water supply [Agency for Toxic Substances and Disease Registry (ATSDR) 1997; Hers et al. 2001; Wu and Schaum 2001].

Toxicologically, TCE is an inherently complex chemical in terms of metabolism, observed effects, and mode of action (MOA), and there is a wide spectrum of views on many scientific issues related to TCE health risks. Consequently, in updating its previous TCE health risk assessments [U.S. Environmental Protection Agency (U.S. EPA) 1985, 1987], the U.S. EPA solicited scientific perspectives from many different groups and individuals. Throughout this scientific outreach effort (e.g., meetings in Williamsburg, Virginia, in 1993 and 1995), the goal of the U.S. EPA was to encourage a diversity of views and encompass a broad range of expertise rather than seek consensus. These efforts culminated in 2000 when, under the sponsorship of the U.S. EPA, the U.S. Air Force, the U.S. Department of Energy, the National Institute of Environmental Health Sciences, and the Halogenated Solvents Industry Alliance, Inc., 16 state-of-the-science (SOS) papers were published as a monograph in a supplemental issue of *Environmental Health Perspectives* (Scott and Cogliano 2000). These papers presented reviews on a range of scientific subjects relevant to TCE health risk assessment, including pharmacokinetics, MOA, epidemiology, and dose–response analysis, and the U.S. EPA drew extensively from them in developing its 2001 draft “Trichloroethylene Health Risk Assessment: Synthesis and Characterization” (U.S. EPA 2001). This draft was subsequently peer reviewed by the U.S. EPA Science Advisory Board (U.S. EPA 2002).

Since that time, substantial new literature has been published relevant to the characterization of TCE hazard and dose response [for a cross-section of recently published research, see U.S. EPA (2004)]. Some of this research is specific to TCE or its metabolites, and some of it describes advances in scientific fields that are more general but that have potential relevance to characterizing the human health risks from TCE. Although some scientific conclusions can be drawn from this updated body of data, speculation as to the impact of these data on the final risk assessment of TCE would be premature at this point, given the ongoing National Academy of Sciences consultation (NAS 2006) and the subsequently planned revision of the U.S. EPA TCE risk assessment. Therefore, the articles in this mini-monograph build on the SOS papers published in 2000 by reviewing recently published literature framed within the context of how it informs a number of key scientific issues believed to be most critical in developing a revised risk assessment. The summaries below briefly describe why these scientific areas are critical to the hazard or dose–response characterization of TCE, how they are or have been scientifically complex or controversial, and recently published scientific literature that may be relevant.

## Key Scientific Issues in TCE and Human Health

### Complex pharmacokinetics

As discussed in Chiu et al. (2006), understanding TCE pharmacokinetics—absorption, distribution, metabolism, and elimination—is critical to both the qualitative and the quantitative assessments of human health risks from environmental exposures. On a qualitative level, pharmacokinetic information can help identify chemical species that may be toxicologically important, by predicting its presence in target tissues. In addition, the delineation of inter-and intraspecies differences can provide insights into how laboratory animal and epidemiologic data may inform overall human health risks and how individuals may differ in their susceptibility. Quantitatively, this information may allow the development of physiologically based pharmacokinetic (PBPK) models to describe the relationship between external measures of exposure and internal measures of toxicologically relevant dose. For TCE, understanding metabolism is particularly important because, as discussed below, for many end points of observed toxicity, the active agent(s) is thought to be a metabolite(s) rather than the parent compound.

As described in Lash et al. (2000a), TCE is a volatile, lipophilic organic solvent that is rapidly absorbed and readily distributed throughout the body. TCE is metabolized primarily by two pathways: oxidation via cytochrome P450s and conjugation with glutathione via glutathione *S*-transferase. There is an extensive database of *in vitro* and *in vivo* studies in both humans and laboratory animals related to TCE pharmacokinetics; these data have been used to varying degrees in the development of PBPK models for TCE and its metabolites. Although the general scheme of TCE metabolism is relatively noncontroversial, the questions of which particular metabolites are formed—in what quantities across species and with what toxicologic significance—are some of the key scientific issues about which there are differing perspectives. Chiu et al. (2006) summarizes some of the outstanding scientific uncertainties in TCE pharmacokinetics and PBPK modeling with a focus on insights that recent data and analyses provide on issues of particular importance to supporting risk assessment. For instance, since 2001, there have been several additional TCE-specific PBPK modeling efforts as well as more general methodologic advances in characterizing PBPK model uncertainty and variability. Chiu et al. (2006) concludes that pending the evaluation of a number of additional structural hypotheses, such as enterohepatic recirculation, plasma binding, and flow- or diffusion-limited treatment of tissue distribution, rigorous application of PBPK modeling to risk assessment appears feasible at least for TCE and its major oxidative metabolites, trichloroacetic acid (TCA) and trichloroethanol. However, there are a number of metabolites of potential toxicologic interest, such as chloral, dichloroacetic acid (DCA), or those derived from glutathione conjugation, for which reliable data are sparse because of analytical difficulties or low concentrations in systemic circulation. Without additional data, it will be a challenge to develop reliable dosimetry for such cases.

### TCE toxicity and MOAs

The articles by Caldwell and Keshava (2006) and Keshava and Caldwell (2006) are discussions of the toxicity of TCE and its metabolites as well as key information related to various MOA hypotheses. As reviewed in the U.S. EPA 2001 draft TCE health risk assessment (U.S. EPA 2001), associations have been reported between TCE and toxicity in a number of organs and tissues. Acute or short-term symptoms of neurotoxicity such as drowsiness, dizziness, and headaches are well established (in fact, TCE was once used as an anesthetic), with a number of studies reporting similar effects with longer-term or chronic exposure (Boyes et al. 2000). Several cancers have been observed in rodent bioassays, including cancers of the liver, kidney, and lung as well as lymphomas, with some related non-cancer effects observed as well. For these cancers, associations have also been reported in epidemiologic studies (Raaschou-Nielsen et al. 2003; Wartenberg et al. 2000b). Good animal models are lacking for other cancers observed in epidemiologic studies. A number of effects in other organs or systems, including the endocrine and reproductive systems and the developing fetus (e.g., cardiac development), have also been reported.

For liver and kidney effects, there are a number of hypothesized MOAs with varying levels of support (Bull 2000; Lash et al. 2000b), whereas for other effects, there is less—or in most cases no—information on MOAs. For liver tumors, proposed MOAs include peroxisome proliferation; oxidative stress; cell-signaling resulting in alterations in cell replication, selection, or apoptosis; and effects on DNA (U.S. EPA 2001). For kidney tumors, they include genotoxicity, accumulation of α_2_μ-globulin, peroxisome proliferation, oxidative stress, nephrotoxicity/cytotoxicity (including the role of formic acid), and the potential role of mutations in the von Hippel-Lindau tumor (VHL) suppressor gene (U.S. EPA 2001).

Part of the reason for so many hypothesized MOAs for TCE toxicity is that exposure to TCE results in a complex internal mixture of parent compound and its metabolites, each of which may act through different MOAs. Thus, understanding which components of this mixture are toxicologically important is a critical step in characterizing the potential human health hazard of TCE. Caldwell and Keshava (2006) review the recent toxicity literature on TCE metabolites with a focus on MOA studies that can particularly help inform TCE toxicity for the liver and kidney. This information not only aids in the identification of the active agent or agents of TCE toxicity (essential for the selection of dose metrics to be used in deriving quantitative estimates of risk) but also provides insights into TCE’s potential MOAs. Moreover, although pharmacokinetic analyses provide substantial information on the prediction of metabolites that circulate at sufficient quantities to be readily detected, the “internal mixture” produced by TCE exposure may contain a number of locally generated or low-concentration metabolites not easily quantified in pharmacokinetic studies but may nonetheless be toxicologically important.

Although much of the attention on liver MOAs has focused on the role of peroxisome proliferator–activated receptor α (PPARα) [discussed in Keshava and Caldwell (2006)], Caldwell and Keshava (2006) discuss a number of other hypotheses for which there is recently published information, such as DNA hypo-methylation. This is important because even if PPARα agonism were to present a plausible MOA, an overriding requirement for reliance on that MOA as a sole indicator of human risk would be that other MOAs have been excluded. This point regarding consideration of alternative hypotheses was emphasized by a recent Federal Insecticide, Fungicide, and Rodenticide Act Scientific Advisory Panel review relating to PPARα (FIFRA SAP 2004) and is a key part of the U.S. EPA cancer guidelines (U.S. EPA 2005).

As mentioned previously, much of the attention on MOA hypotheses for TCE toxicity has focused on the role of PPARα, the subject of the article by Keshava and Caldwell (2006). Although there has been only limited research on PPARα and TCE specifically, a vast literature has emerged on PPARs more generally in the last 4 or 5 years [a search of the PubMed database (http://www.ncbi.nlm.nih.gov/entrez/query.fcgi) on 20 September 2005 for “peroxisome proliferator-activated receptor” returned 3,495 entries published since 2001]. Given this new information, particularly with respect to the pleiotropic nature of PPARα-related effects, Keshava and Caldwell (2006) discuss various recent hypotheses about the MOA or MOAs for PPAR agonist–related tumorigenesis.

The issue of whether PPARα agonists pose human health risks is controversial, both in the case of TCE and in general. TCE and two of its key metabolites, TCA and DCA, have been characterized as weak peroxisome proliferators. In the last 10 years, there has been a considerable evolution in the hypotheses about the relationship between PPARα agonism and tumor induction in rodents. Initially, it was hypothesized that the proliferation of peroxisomes themselves was causally related to cancer (e.g., Cattley et al. 1998), but today most investigators believe the frank effect is, at best, associative (Klaunig et al. 2003; Melnick 2001). Part of the controversy, therefore, stems from a matter of interpretation, particularly the degree to which inferences should be made about human risk on the basis of such an association.

Another point of controversy stems from the question of how sensitive are humans to events causally related to carcinogenesis. It is clear now that PPARα can be activated in humans and that humans are at least as sensitive as laboratory animals for some effects, that is, those related to lipid regulation and that are the target of an extensive effort to develop pharmaceuticals using this mechanism. Characterizing the effects from PPARα agonism in the identification of key events that may constitute a cogent MOA hypothesis for tumor induction has yet to be resolved. Moreover, concerns have been raised about the adequacy of human data to determine the carcinogenic potential in humans for drugs that cause peroxisome proliferation in rodents (FIFRA SAP 2004; Melnick 2001; Newman and Hulley 1996). For all these issues, an examination of the full spectrum of pleiotropic responses to PPARα agonism—particularly extraperoxisomal effects and their relationships with PPARα-independent processes—is needed. Keshava and Caldwell (2006) review this spectrum of effects with a focus on recently published data.

### Epidemiologic studies

The final article by Scott and Chiu (2006) discusses TCE cancer epidemiology. Because epidemiology is based on human data, it is a key element to the hazard characterization of TCE health risks. Epidemiologic evidence on occupational and residential exposures can contribute information regarding associations between exposure and cancer. Well-conducted epidemiologic studies that show a positive association between an agent and a disease are accepted as the most convincing evidence about human risk (National Research Council 1983).

More than 80 epidemiologic studies and reports have assessed possible associations between TCE exposure and various health outcomes. Many of these studies have examined cancer mortality; fewer have examined cancer incidence. Wartenberg et al. (2000b), as part of the SOS papers, developed a “joint analysis” of the cancer epidemiology studies. Cohort studies were grouped according to the specificity of the exposure assessment and the study results were combined by weighting by the inverse variance, whereas case–control and community studies were systematically reviewed. Wartenberg et al. (2000b) concluded that the epidemiologic evidence supported an association between TCE exposure and liver cancer, kidney cancer, and lymphomas. Somatic mutations of the VHL tumor suppressor gene have been found in kidney tumors of TCE-exposed workers (Brauch et al. 1999; Brüning et al. 1997). Furthermore, these findings may inform weight of evidence evaluations of the epidemiologic observations.

The Wartenberg et al. (2000b) analysis was not without controversy, as it was the subject of a number of letters to the editor (e.g., Borak et al. 2000; Wartenberg et al. 2000a) and several public comments on the U.S. EPA draft assessment. Many of the comments focused on how the studies were grouped or how the study results were combined. In addition, alternative meta-analyses and interpretations of the epidemiologic evidence have been published or presented since 2000 (e.g., Mandel and Kelsh 2001).

Cancers of the liver, kidney, and lymphatic system are of low incidence [≥2% lifetime risk of diagnosis, Ries et al. (2005)] and are nonspecific to TCE, which partly contributes to the inconsistent observations in the body of epidemiologic evidence. Other factors have been identified or suggested as associated with these cancers, limiting the statistical power of individual studies. Additionally, mean exposure concentrations in TCE cohort studies are relatively low, with an impact of further limiting statistical power. Lower exposure levels are not likely to produce a large magnitude of risk. The epidemiologic picture for TCE differs greatly from that for the known human carcinogen vinyl chloride, for example, where angiosarcoma is specific to this exposure and exposure concentrations in the epidemiologic studies are much higher than the concentrations reported for the TCE cohorts (Kielhorn et al. 2000).

In this light, an interpretation of the database of cancer epidemiology needs to take into consideration a number of aspects, the subject of the article by Scott and Chiu (2006). They first provide an update on the epidemiologic studies that have been published since the Wartenberg et al. (2000b) review, including a recent study assessing mutations in the VHL tumor suppressor gene. Although recently published studies appear to provide further support for the kidney, liver, and lymphatic systems as targets of TCE cancer toxicity, Scott and Chiu note that a number of challenging issues need to considered before drawing causal conclusions as to TCE exposure and cancer.

Some of the important factors discussed by Scott and Chiu (2006) include weighing the strengths and limitations of various study types, and characteristics include study differences in exposure characterization and the relative sensitivity of mortality versus incidence data. Such differences may contribute to false-positive or false-negative observations and need to be factored into any overall characterization. In addition, issues surrounding lymphomas warrant further discussion. More recent epidemiologic studies of TCE exposure observe associations with lymphoma, particularly non-Hodgkin lymphoma (NHL) (Hansen et al. 2001; Raaschou-Nielsen et al. 2003; Wartenberg and Scott 2002). Evaluating associations with malignant lymphomas poses particular challenges because they include a diverse group of diseases such as NHL, Hodgkin disease, multiple myeloma, and leukemia. NHL incidence has been increasing and is now the sixth leading cause of cancer deaths in males in the United States (Fisher 2003). The classification of lymphoid neoplasms, specifically lymphomas, has recently undergone a substantial revision, primarily on the basis of new findings from molecular biology, genetics, and immunology (Herrinton 1998). For this reason, Scott and Chiu (2006) note that inconsistent observations in the epidemiologic studies for different categories of lymphoid tumors may not be inconsistent with emerging data on their pathogenesis. Moreover, a better mechanistic understanding of this disease may help clarify how these tumors are associated with TCE exposure.

## Next Steps in TCE Risk Assessment

This mini-monograph does not and could not address all the issues related to the potential health effects of environmental exposure to TCE that need to be addressed as the U.S. EPA revises its assessment, but we believe it does provide updated perspectives on some of the more critical and contentious scientific issues. More generally, given the complexity, uncertainty, and varying perspectives on these and other important scientific issues, a federal interagency working group coordinated by the White House Office of Science and Technology Policy (OSTP) decided that a scientific consultation with an NAS panel would be beneficial and informative to clarify the SOS as the U.S. EPA moves forward in completing its health risk assessment. This consultation was initiated in September 2004, coordinated by the OSTP and co-sponsored by a number of other federal agencies, including the U.S. EPA, the U.S. Department of Defense, the U.S. Department of Energy, and the National Aeronautics and Space Administration. The charge to the NAS is provided in the Appendix, and additional information on this consultation is available from the NAS website (NAS 2006). A report from the NAS is expected in 2006.

The advice from the NAS on these and other scientific issues, together with comments already received from the Science Advisory Board and the public and recently published scientific literature, will be incorporated into a revised U.S. EPA risk assessment of TCE. Because of the substantial amount of new information and analysis that is expected, the revised draft of the assessment will undergo further peer review and public comment before completion. We believe that through this process, the U.S. EPA risk assessment will reflect the SOS on the human health effects of TCE.

## Appendix: Charge to the National Academy of Sciences Committee on Assessing the Human Health Risks of Trichloroethylene: Key Scientific Issues.^a^

The NAS panel of experts shall use their best scientific judgment to provide advice on critical underlying specific scientific issues related to the assessment of the potential human health risks from environmental exposure to TCE. The panel shall strive to reach a consensus on all critical issues.

In providing its advice and review, the NAS panel shall highlight issues critical to the development of an objective, realistic, and scientifically balanced TCE health risk assessment. In doing so, the panel should distinguish between issues that can be addressed through short-term analyses and issues that are more appropriately addressed through medium- or long-term research projects. Special attention should be given to the availability of appropriate data and methods to implement the panel’s advice, as well as the distinction between data analysis and data generation.

The following outlines the scientific issues upon which to focus the evaluation:

### Hazard Characterization/Mode of Action

Key scientific issues regarding various alternative hypotheses as to the MOAs for TCE toxicity and their relevance to humans include the following:

- Liver tumors: Production and toxicological importance of metabolites TCA and DCA in rodents and humans; strength of evidence for various hypothesized MOAs, including peroxisome proliferation; cell signaling resulting in alterations in cell replication, selection, and/or apoptosis; and effects on DNA.
- Kidney tumors: Production and toxicologic importance of various glutathione *S*-transferase–pathway metabolites such as DCVG [*S*-(1,2-dichlorovinyl)glutathione] and DCVC [*S*-(1,2-dichlorovinyl)-l-cysteine] in rats and humans; strength of evidence for various hypothesized MOAs, including genotoxicity; accumulation of α_2_μ-globulin; peroxisome proliferation; nephrotoxicity/cytotoxicity (including role of formic acid).
- Strength of evidence for various hypothesized mode(s) of action for other effects (e.g., neurotoxicity; noncancer liver and kidney toxicity; immunotoxicity and lymphoid cancer; developmental, reproductive, and endocrine effects; pulmonary effects and lung cancer).
- Identification of key events in modes of action.
- Potential for contributions from multiple modes of action.
- Dose-dependent differences in MOAs.

Key scientific issues regarding possible approaches to synthesize epidemiologic data in informing the hazard characterization of TCE include the following:

- Use of meta-analytical techniques to summarize epidemiologic findings, including methods for classifying and weighting studies.
- Strengths and limitations of the body of epidemiologic evidence with respect to kidney cancer.
- Strength of evidence provided by reported molecular information on associations with mutations in the von Hippel-Landau tumor suppressor gene.
- Identification of key epidemiologic studies for informing overall conclusions on TCE potential carcinogenicity.

Key scientific issues regarding the potential for differential susceptibility to TCE’s toxic effects in different subpopulations or life stages include the following:

- Effects of altered metabolism, whether by intrinsic or acquired factors, and/or cumulative exposures on the toxicologically important internal doses of TCE and its metabolites.
- Pharmacodynamic factors such as genetic polymorphism, disease states, and developmental windows as they relate to TCE toxicity.
- Degree to which differential susceptibility can be quantified.

### Physiologically Based Pharmacokinetic Modeling

Key scientific issues regarding approaches for pharmacokinetic modeling based on existing metabolic information of TCE include the following:

- Relative strengths and limitations of different model structures and parameterizations, including the tradeoff between model complexity (and hence completeness) and uncertainty.
- Evaluation of model uncertainties, including the use of Markov-Chain Monte Carlo methods.

Key scientific issues regarding the uses of pharmacokinetic modeling results for risk assessment of TCE include the following:

- Dose metrics [e.g., TCA AUC (area under the curve), DCA AUC in blood and/or tissues] for developing human equivalent doses, route-to-route extrapolations, or use in biologically based dose–response modeling.
- Addressing uncertainties associated with PBPK-based dose-metrics, including the consideration of non-PBPK-based scaling approaches.
- Assessment of the impact of human variation and other factors such as enzyme induction or chemical mixtures on toxicologically important doses.

### Dose–Response Assessment

Key scientific issues regarding the quantitative assessment of non-cancer risks include the following:

- Strengths and limitations of studies to consider in quantifying non-cancer risks, including consideration of studies in various species (including humans), of various durations (acute, short term, chronic), and with various observed end points (e.g., liver weight changes, cardiac anomalies in prenatal development, renal effects, endocrine effects, and reproductive effects).
- Approaches to develop point(s) of departure, including use of PBPK-based human equivalent doses and benchmark dose or other modeling.
- Use of PBPK-based Monte Carlo analysis quantify human variation.

Key scientific issues regarding the quantitative assessment of cancer risks include the following:

- Strengths and limitations of studies—including both rodent studies and epidemiologic studies—to consider in quantifying cancer risks, including consideration the uncertainty in exposure estimates, MOA, and species extrapolation.
- Quantitative approaches to use in estimating risk, including PBPK-based dose estimates, linear and nonlinear extrapolation, and biologically based dose–response modeling.
- Methods to characterize the uncertainty and/or variability in estimates of cancer risk, including consideration of both statistical (possibly Bayesian) and nonstatistical approaches and of the potential for differential susceptibility among certain subpopulations.
